# Facile Mixing of Phospholipids Promotes Self-Assembly of Low-Molecular-Weight Biodegradable Block Co-Polymers into Functional Vesicular Architectures

**DOI:** 10.3390/polym12040979

**Published:** 2020-04-22

**Authors:** Amit Kumar Khan, James C. S. Ho, Susmita Roy, Bo Liedberg, Madhavan Nallani

**Affiliations:** 1 Centre for Biomimetic Sensor Science, School of Materials Science and Engineering, Nanyang Technological University, 50 Nanyang Drive, Singapore 637553, Singapore; akhan@acmbiolabs.com (A.K.K.); james.hcs@ntu.edu.sg (J.C.S.H.); susmita.chemistry@gmail.com (S.R.); bliedberg@ntu.edu.sg (B.L.); 2 ACM Biolabs Pte. Ltd., NTU Innovation Center, 71 Nanyang Drive, Singapore 638075, Singapore

**Keywords:** self-assembly, polymer-lipid hybrid, biodegradable polymer, polymersome, giant unilamellar vesicles, phospholipase A_2_

## Abstract

In this work, we have used low-molecular-weight (PEG_12_-*b*-PCL_6_, PEG_12_-*b*-PCL_9_ or PEG_16_-*b*-PLA_38_; M_W_, 1.25–3.45 kDa) biodegradable block co-polymers to construct nano- and micron-scaled hybrid (polymer/lipid) vesicles, by solvent dispersion and electroformation methods, respectively. The hybrid vesicles exhibit physical properties (size, bilayer thickness and small molecule encapsulation) of a vesicular boundary, confirmed by cryogenic transmission electron microscopy, calcein leakage assay and dynamic light scattering. Importantly, we find that these low M_W_ polymers, on their own, do not self-assemble into polymersomes at nano and micron scales. Using giant unilamellar vesicles (GUVs) model, their surface topographies are homogeneous, independent of cholesterol, suggesting more energetically favorable mixing of lipid and polymer. Despite this mixed topography with a bilayer thickness similar to that of a lipid bilayer, variation in surface topology is demonstrated using the interfacial sensitive phospholipase A_2_ (sPLA_2_). The biodegradable hybrid vesicles are less sensitive to the phospholipase digestion, reminiscent of PEGylated vesicles, and the degree of sensitivity is polymer-dependent, implying that the nano-scale surface topology can further be tuned by its chemical composition. Our results reveal and emphasize the role of phospholipids in promoting low M_W_ polymers for spontaneous vesicular self-assembly, generating a functional hybrid lipid-polymer interface.

## 1. Introduction

Nature has evolved a self-assembled lipid membrane that encloses a crowded pool of proteinaceous constituents, allowing the selective exchange of information between the intracellular milieu and the extracellular environment [1,2]. The structural integrity of the native membrane is dependent on the chemical composition and properties of its constituents including the acyl chain lengths, degrees of unsaturation [3,4], polar headgroup charges, chemical structures and constituent protein entities [5,6]. When removed from its native environment, the membrane is susceptible to chemical modifications [7] and physical perturbations [8,9]. Bearing structural homology to lipids, synthetic block copolymers (BCPs) have emerged as alternatives, with enhanced stability and highly tunable interfacial properties [10,11], including the ability to tune membrane thickness and introduce functional groups for selective chemical modification making it a versatile system.

Mixing liposomal and polymeric materials to fabricate hybrid lipid-polymer vesicles integrates individual strengths of the self-assembling lipid and polymer building blocks, and has been extensively studied [12,13]. In past decades, liposomes have been widely exploited in the pharmaceutical and cosmetic industries as carriers, due to their high biocompatibility, albeit lower stability and shelf life [14,15]. Polymersomes, on the other hand, show higher mechanical and chemical stability and biofunctionality [16]. However, they suffer from lower biocompatibility and, therefore, have not been as widely applied as the lipid counterpart. A mixed system allows one to obtain a vehicle that is robust (mechanically stable with low permeability) [17], chemically versatile (possibility to be tuned and functionalized), and biocompatible, thus providing a promising avenue for diverse applications. Indeed, this has been explored in recent years using different di- and tri-block copolymers, in combination with lipids forming fluid or gel phases [13,18,19]. 

In hybrid vesicles, BCPs and lipids display two distinct lateral membrane topologies—homogeneous or phase-separated of which the latter is characterized by co-existing segregated domains. Domain formation is controlled by an interplay of multiple parameters [20], including but not limited to: lipid phase, thickness mismatch [21,22], lipid/polymer ratio, cholesterol [23], and protein–lipid crosslinking [24]. Given the high compositional degrees of freedom for polymer chemical architectures and structural adaptation for specific geometry and dynamic requirement of the bilayer, domain formation is not entirely understood yet. Nonetheless, several denominating conditions for promoting domain formation are becoming clear: the existence of lipid gel phase below the gel–liquid–crystalline transition temperature (T_m_) and increased hydrophobic thickness mismatch in the presence of cholesterol. Compositions promoting the formation of de-mixed lipid-rich and polymer-rich phases are demonstrated by PEG_13_-*b*-PBD_22_:DPPC and PEG_13_-*b*-PBD_22_:POPC:Chol (1:1:1), at room temperature [23]. These domains persist in both micron- and nano-sized vesicles, at a comparable domain size-to-vesicle size ratio [25]. Some higher molecular weight polymer (≥3800 g mol^−1^) have been reported to form a homogeneous surface [23,24,26,27] and it has been reasoned that it is due to equilibration of a transient phase separated topology, arises due to hydrophobic mismatch [20].

Incorporation of biodegradable polymer with phospholipids [28,29,30,31,32] to form nano-scale, and only one example of micron-scale biodegradable hybrid vesicles [28] have been reported. For nano-sized hybrid vesicle formation, mixing of up to 10 mol% of polyethyleneglycol-polycaprolactone (PEG-*b*-PCL) copolymers (7500–10,600 g·mol^−1^) with 1,2-dipalmitoyl-sn-glycero-3-phosphocholine (DPPC) has been reported [29]. A similar compositional range used for PEGylated lipids in a stealth liposome formulations [30]. M_W_ of these polymers are comparatively larger (10 times or more) than phospholipid with *f_hydrophilic_* between 47–70% by the mass of BCPs. An interesting hybrid vesicle formed from DPPC lipid and triblock copolymers (PCL_12_-*b*-PEO_45_-*b*-PCL_12_, M_W_: 5282 g mol^−1^, and PCL_16_-*b*-PEO_104_-*b*-PCL_16_, M_W_ 14,375 g mol^−1^) demonstrated that both copolymers increased the thermal stability of the lipid bilayer while decreased the water outflux and release of calcein from the inner part of liposome [31]. Another study suggested that the incorporation of high M_W_ PEG-*b*-PCL (10,600 g mol^−1^) in Tween 80/cholesterol Niosome vesicles resulted in spherical and wormlike micelles coexist with the vesicle. The morphologies are explained by the difference of hydrophilic/hydrophobic ratio, molecular size, mass ratio, and possibly phase segregation between BCP and lipid within the nanostructures [32]. In consideration of these factors, designing and synthesizing hybrid vesicles with BCPs of comparable length may be energetically more favorable. 

**Scheme 1:**
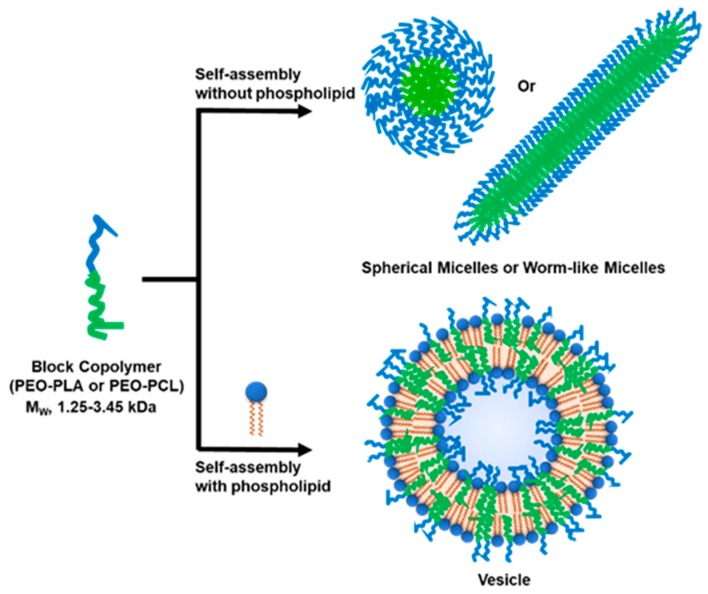
Self-assembly of low-molecular-weight block copolymers in the absence and presence of phospholipid.

Hence, in this work, low-molecular-weight (M_W_) biodegradable BCPs (1.25–3.45 kDa), which are predicted to give rise to minimal thickness mismatch in the hybrid bilayer, were selected. This is in contrast to the use of higher M_W_ BCPs (≥3800 g/mol) to construct hybrid vesicles assemblies [23,24,26,27,28,29,30,31,32]. These low M_W_ biodegradable BCPs are relatively unexplored [33] and, therefore, not known to form vesicular assemblies. We hypothesize that these BCPs will self-assemble with lipids in a more energetically favorable configuration, better mimicking the topology of a lipid membrane, including a semipermeable vesicular boundary, lipid-like membrane thickness, and functional response to interfacial-sensitive enzymatic activity (Scheme 1). We have constructed hybrid vesicles composed of a fluid phase lipid, 1-palmitoyl-2-oleoyl-*sn*-glycero-3-phosphocholine (POPC) and a biodegradable BCP (PEG_12_-*b*-PCL_6_, PEG_12_-*b*-PCL_9_ or polyethyleneglycol_16_-*b*-polylactic acid_38_ (PEG_16_-*b*-PLA_38_) at equimolar composition, spanning the nano- and micron-scales. We have previously reported that an equimolar mixture of PEG_13_-*b*-PBD_22_ and POPC gives rise to tubular vesicles earlier [34] and hence used it for comparison. The hybrid vesicles were characterized by cryogenic-transmission electron microscopy (cryo-TEM), dynamic light scattering (DLS), fluorescence microscopy and calcein leakage assay for their topology and morphology. Furthermore, to assess the bio-functional characteristics of such hybrid vesicles, they were subjected to an interfacial-dependent phospholipase digestion assay. 

We present the following results: First, phospholipids actively promote the self-assembly of vesicular architectures of the selected BCPs to readily form vesicles at the nano- and micron-scales—a role not previously emphasized. These polymers do not form well defined polymer vesicles in the absence of the phospholipid. Second, the hybrid vesicles exhibit a homogeneous bilayer thickness similar to that of a lipid vesicle, consistent with the low M_W_ property of the selected BCPs. Third, the ability to form stable homogeneous large-(LUV) and giant unilamellar vesicles (GUV) is an indication that hydrophobic mismatch is below a phase-separating threshold within the range of vesicle size explored, which further supports the notion that there is mutual compatibility between the polymer and lipid. Fourth, the appearance of phase-separated topology does not follow a simple correlation with known domain-forming conditions [23]. Rather, it is likely dependent on the specific chemical nature of the polymer [16,35]. Finally, we show that the sensitivity of the nano-sized hybrid vesicles toward an interfacial-dependent phospholipase can be modulated, raising the possibility that the surface topography of these hybrid vesicles can be controlled simply by tuning the chemical composition of the BCPs. 

## 2. Materials and Methods

### 2.1. Materials

PEG_12_-*b*-PCL_6_, PEG_12_-*b*-PCL_9_, PEG_16_-*b*-PLA_38_, and PEG_13_-*b*-PBD_22_ were purchased from Polymer Sources Inc., Quebec, Canada. 1-palmitoyl-2-oleoyl-*sn*-glycero-3-phosphocholine (POPC), 1,2-dipalmitoyl-*sn*-glycero-3-phosphoethanolamine-*N*-(lissamine rhodamine B sulfonyl) (Rhod-DPPE), 1,2-dipalmitoyl-sn-glycero-3-phosphoethanolamine-N-(7-nitro-2-1,3-benzoxadiazol-4-yl) (NBD-DPPE) and cholesterol were from Avanti Polar Lipids, Alabaster, AL, USA. Phospholipase A_2_ (P9279) and Naphtho[2,3-α]pyrene (380946) was from Sigma-Aldrich, Singapore. Triton X-100 was from BioRad, Singapore. Calcein from TCI Chemical, Singapore. Phosphate-buffered saline (PBS, 10×) solution was purchased from Gibco, Singapore. All other chemicals were purchased from Sigma-Aldrich (St. Louis, MO, USA) unless stated.

### 2.2. Preparation of Large Unilamellar Vesicles (LUVs)

LUVs were prepared by the solvent dispersion method, followed by extrusion. 200 mg/mL stock solutions of POPC and polymer were prepared by dissolving solid POPC and polymer in tetrahydrofuran (THF). 0.5 equivalent (2.5 µmol) of POPC stock solution and 0.5 equivalent (2.5 µmol) of polymer stock solution mixed in a small 2 mL glass vial and vortexed to prepare Solution A (for a POPC liposome, 1.0 equivalent (5 µmol) of POPC stock solution was taken). After mixing, Solution A was aspirated in a 50 µL Hamilton glass syringe. 1 mL 1× PBS, pH 7.4, was taken in a 5 mL small glass test tube (Solution B). Solution A was added slowly to 1 mL 1× PBS (Solution B) while vortexing (600–700 rpm) at room temperature. A turbid solution was obtained. For calcein-encapsulated LUVs, 1 mL of 30 mM calcein in PBS was used. The resultant solution was extruded 21 times through a 200 nm membrane filter (Avanti Polar Lipid Inc., Alabaster, AL, USA) using a 1 mL mini-extruder (Avanti Polar Lipid Inc., Alabaster, AL, USA) to get monodispersed LUVs. Unencapsulated calcein was removed by overnight dialysis using 300 kDa regenerated cellulose membrane against PBS.

### 2.3. Dynamic Light Scattering (DLS)

DLS was performed on Malvern Zetasizer Nano ZS (Malvern, UK). 100 µL of the 10-fold diluted, purified, filtered sample was placed in a micro cuvette (Eppendorf^®^ UVette, Sigma-Aldrich, Singapore) and an average of 30 runs (10 s per run) was collected using the 173° detector. Data was plotted in Microsoft Excel (Microsoft, Redmond, WA, USA)

### 2.4. Cryogenic-Transmission Electron Microscopy (Cryo-TEM)

For cryo-TEM, 4 µL of the sample containing POPC/BCP hybrid vesicles (5 mg/mL) were adsorbed onto a lacey holey carbon-coated Cu grid, 200 mesh size (Quantifoil, Großlöbichau, Germany). The grid was surface treated for 20 s using glow discharge before use. After adding the sample, the grid was blotted with Whatman filter paper (GE Healthcare Bio-Sciences, Piscataway, NJ, USA) for 2 s, with blot force 1, and then was plunged into liquid ethane at −178 °C using Vitrobot (FEI Europe B.V., Eindhoven, The Netherlands). The cryo-grids were imaged using a FEG 200 keV transmission electron microscope (Arctica, FEI Europe B.V., Eindhoven, The Netherlands) equipped with a direct electron detector (Falcon II, Fei Europe B.V., Eindhoven, The Netherlands). Images were analyzed in ImageJ.

### 2.5. Calcein Leakage Assay

Dialyzed calcein encapsulated polymer/lipid hybrid LUVs were 100-fold diluted with PBS before leakage experiment. 100 µL of the diluted calcein encapsulated samples were added to a 96-well flat black plate (Corning Incorporated, Corning, NY, USA). Fluorescence was measured at 520 nm (excitation at 480 nm) using a fluorescence plate reader (Infinite 200 Pro, Tecan, Salzburg, Austria). 5 µL of 1% Triton X-100 was added for total lysis of the vesicles. In order to estimate the encapsulation of calcein for each formulation, fluorescence of calcein of dialyzed hybrid vesicles as background was subtracted from that of lysed hybrid vesicles with Triton X-100.

### 2.6. Preparation of Giant Unilamellar Vesicles (GUVs)

GUVs were prepared by the electroformation method [36]. Briefly, 20 µL of a 1 mg/mL POPC or POPC/BCP mixture at 1:1 molar ratio (or 1:1:1 for POPC/BCP/cholesterol blend) containing either 1% of Rhod-DPPE (or 0.5% Rhod-DPPE and 1.0–1.5% naphthopyrene) in CHCl_3_ was spread and air dried on two Indium tin oxide (ITO)-coated glass slide (Nanion Technologies GmbH, Munich, Germany) using a glass syringe to form a thin POPC/BCP film. The film was further dried for another 1 h under vacuum. Subsequently, an “O” ring was placed on one ITO-coated glass slide. Then, 250 µL of 300 mM sucrose solution was added to the “O” ring enclosed area and sandwiched by a second ITO-coated glass slide. A current was applied at 3 V, 5 Hz, for 120 min at 50 °C and the formed GUVs were collected and used immediately or stored at 4 °C for up to a week.

### 2.7. Wide-Field Deconvolution Microscopy

A DeltaVision microscope (Applied Precision Inc., Issaquah, WA, USA), fitted with a PLAPON 60XO/1.42 NA oil-immersion macro apochromat objective from Olympus and tetramethylrhodamine-isothiocyanate (TRITC) and fluorescein-isothiocyanate (FITC) Semrock filters (New York, NY, USA), was used for imaging GUVs in real time. Samples were imaged in a 96-well uncoated μ-plate with a glass bottom (ibidi GmbH, Gräfelfing, Germany). Briefly, 5–10 µL of sucrose-encapsulating GUVs were added into 200 μL of osmotically balanced 300 mM glucose solution. The sucrose-loaded GUVs settled to the bottom of the well within 1–2 min and were imaged using TRITC and/or FITC filter sets. 

### 2.8. Phospholipase A_2_ Enzymatic Assay

For phospholipase A_2_ (sPLA_2_) assay, 50 μL of dialyzed calcein-encapsulating LUVs (1:50 diluted in PBS) were mixed with 50 μL of PBS containing 1 mM of CaCl_2_ and different concentrations of sPLA_2_ (10–400 nM). The release of calcein from the vesicles, as result of the addition of sPLA_2_, was monitored for 60 min (at 30 s intervals) at room temperature using a fluorescence plate reader (Infinite 200, Tecan, Salzburg, Austria; λex = 480 nm, λem = 520 nm). The dialyzed vesicles were used within a week. The percentage of calcein release over time is calculated as above. Percentage leakage was calculated based on the following equation: 100 × (*F_t_ − F*_0_)/(*F*_T_ − *F*_0_), where *F*_0_ is the initial fluorescence of calcein, *F_t_* is the fluorescence of calcein at time interval *t* and *F*_T_ represents total fluorescence, i.e., complete release of calcein upon addition of 0.05% Triton X-100. For GUVs, 5–10 μL of the GUV solution was added to a prediluted 100 nM sPLA_2_ solution (in 300 mM glucose for osmotic equilibrium), supplemented with 200 nM CaCl_2_.

## 3. Results and Discussion

### 3.1. Selection of Block Co-Polymers (BCPs)

In this study, we have chosen three low M_W_ biodegradable BCPs with varying M_W_ (1.25–3.45 kDa) and the hydrophilic block fraction, *f_hydrophilic_* (0.20–0.44). They are PEG_12_-*b*-PCL_6_, PEG_12_-*b*-PCL_9_, and PEG_16_-*b*-PLA_38_, abbreviated as PCL_0.7_, PCL_1.1_, and PLA_2.75_, respectively (Table 1). Additionally, we have included a fully synthetic non-biodegradable PEG_13_-*b*-PBD_22_, abbreviated as PBD_1.2_. Self-assembly of BCPs into vesicles is mainly defined by their *f_hydrophilic_* and ε_h_, the monomer’s effective interaction energy with the bulk solution [16,37]. Since the *f_hydrophilic_* values are within the theoretical boundary for vesicle assembly, the propensity to form vesicles will likely be thermodynamically constrained by the effective aggregate stability [16]. 

### 3.2. Formation of Hybrid POPC/BCP Large Unilamellar Vesicles

**Figure 1:**
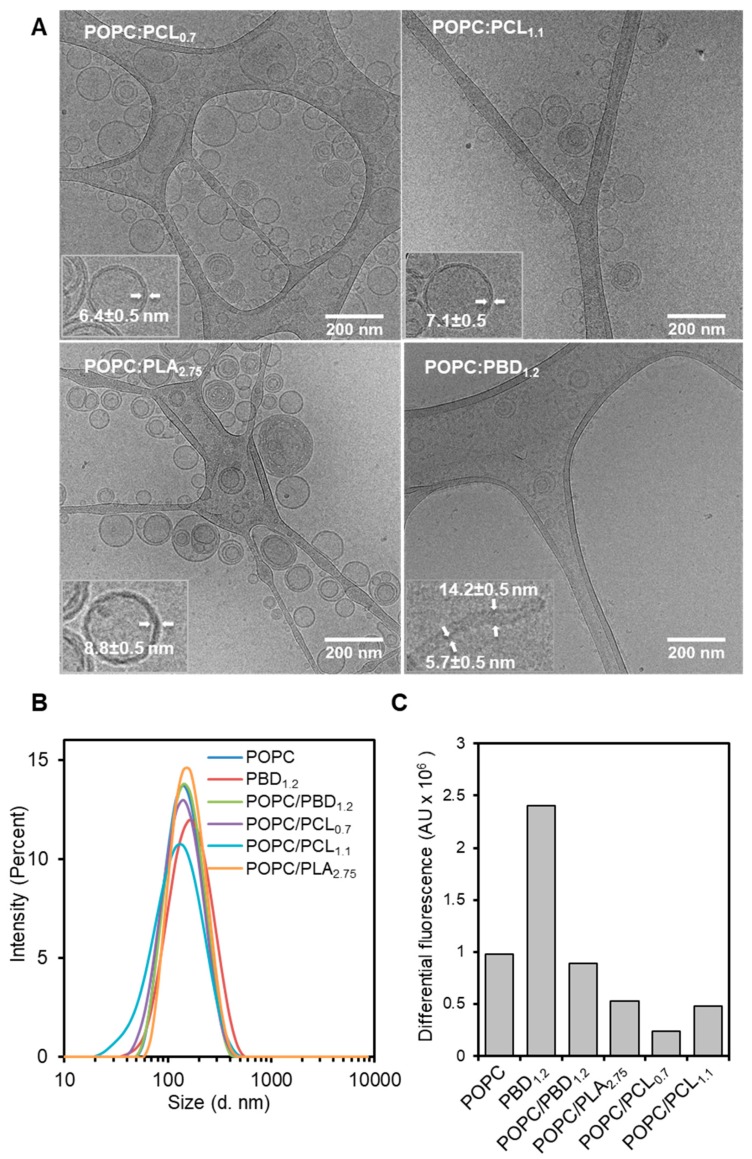
Molecular assemblies of hybrid vesicles. (**A**) Cryogenic-transmission electron microscopy (cryo-TEM) images of hybrid vesicles formed by solvent dispersion and extrusion method in phosphate-buffered saline (PBS). Average membrane thickness is indicated in the bottom left inset of each image; (**B**) Intensity-weighted hydrodynamic diameter of hybrid vesicles; and (**C**) Differential Fluorescence of Calcein of lysed hybrid vesicles with Triton X-100 after background subtraction of dialyzed hybrid vesicles.

BCPs or POPC/BCPs mixtures at equimolar ratios were used to prepare large unilamellar vesicles (LUVs) by the solvent dispersion and extrusion method, followed by morphological characterization using cryogenic-transmission electron microscopy (cryo-TEM) and dynamic light scattering (DLS). The electron micrographs showed a radially uniform density distribution as well as the Fresnel interference fringes on the inner and outer membrane edges, suggesting topographically uniform vesicles were obtained [34] (Figure 1A). Line profile measurements estimate the bilayer thicknesses of 6.4 ± 0.6 nm, 7.1 ± 0.6, 8.8 ± 0.6, and 5.7 ± 0.6 nm for POPC/PCL_0.7_, POPC/PCL_1.1,_ POPC/PLA_2.75_, and POPC/PBD_1.2_, respectively. For POPC, 5.3 ± 0.5 nm was obtained. For POPC/PBD_1.2_, in addition to the vesicular structure, cylindrical tubes with a diameter of about 14 nm were also observed, which is consistent with previous studies [34]. A closer inspection on the density distribution reveals a clear midplane of low density for POPC/PCL_0.7_ and POPC/PCL_1.1_ that is less distinct for POPC/PLA_2.75_, suggesting chain overlap (interdigitation) as well as chain entanglement. In contrast, polydisperse and non-uniform assemblies were observed for the BCPs (Figure S1). PCL_0.7_ and PCL_1.1_ formed worm-like micellar structures with a diameter of about 12 nm (Figure S1A,B), whereas PLA_2.75_ formed polydisperse assemblies comprising of micelles and vesicle-like morphology with a membrane thickness of about 17 nm (Figure S1C). 

The size estimations of the hybrid vesicles by DLS showed unimodal intensity-weighted distribution with a mean z-average hydrodynamic diameter, D_h_, of 135 ± 12 nm, and PdI values ranges between 0.12–0.21 (Figure 1B). The size of the vesicles was comparable (POPC, 135 nm; PBD_1.2_, 146 nm; POPC/PBD_1.2_, 132 nm; POPC/PCL_0.7_, 123 nm; and POPC/PLA_2.75_, 147 nm), except POPC/PCL_1.1_ (108 nm), which showed a much smaller size. 

We further determined if the hybrid vesicles maintained a closed shell by preparing hybrid LUVs in buffer containing calcein at its self-quenching concentration (30 mM). For hybrid vesicles, the total molar mass of POPC and BCP was kept constant to that of POPC preparation so that a comparable amount of vesicles was prepared for each sample. Total encapsulated calcein was determined by vesicle lysis with 0.05% Triton X-100, after removal of unencapsulated calcein by dialysis (Figure 1C). The result shows that, PBD_1.2_ vesicle has much higher amount encapsulated compared to POPC. The amount of encapsulation for the hybrid POPC/PBD_1.2_ was reduced to a level similar to that of POPC. In comparison, all the hybrid biodegradable vesicles showed further reduction in the absolute increase in fluorescence intensity (POPC/PLA_2.75_ > POPC/PCL_1.1_ > POPC/PCL_0.7_) after prolonged dialysis time of 48 h (Figure 1C and Figure S2). This suggests that the hybrid biodegradable vesicles are partially leaky to calcein, based on the current self-assembly protocol, which has been previously optimized for POPC, PBD_1.2_, and POPC/PBD_1.2_ [34]. In contrast, the biodegradable BCPs alone did not show encapsulation.

Corroborating with the cryo-TEM, DLS, and leakage assay results, it is clear that POPC promotes self-assembly of biodegradable low M_W_ BCPs into hybrid LUVs with similar structural properties (hydrodynamic size and bilayer thickness), but varying functional capability (encapsulation), due likely to the compatible physicochemical properties of the lipid and BCPs, as well as the chemical diversity of the BCPs. The results are striking in view of the general conception that low M_W_ BCPs are not known to microphase separate presumably due to lower monomer’s effective interaction energy [16]. Hydrophobicity of the PBD, PLA, and PCL blocks differ and play an important role in interaction energy driven self-assembly [16,38]. The presence of oxygen atoms in the hydrophobic building block makes PLA and PCL less hydrophobic compared to inert PBD. Furthermore, the same molecular weight PLA contains more oxygen atom (one building block containing three carbon and two oxygen) than PCL (six carbon and two oxygen). Therefore, PCL is more hydrophobic than PLA. Thus, the order of hydrophobicity is PBD > PCL > PLA. As a result, to form polymeric vesicles, PLA and PCL require lower *f_hydrophilic_* (~20%) and higher molecular weight BCP [16]. 

### 3.3. Surface Topography of POPC/BCP Giant Unilamellar Vesicles

**Figure 2:**
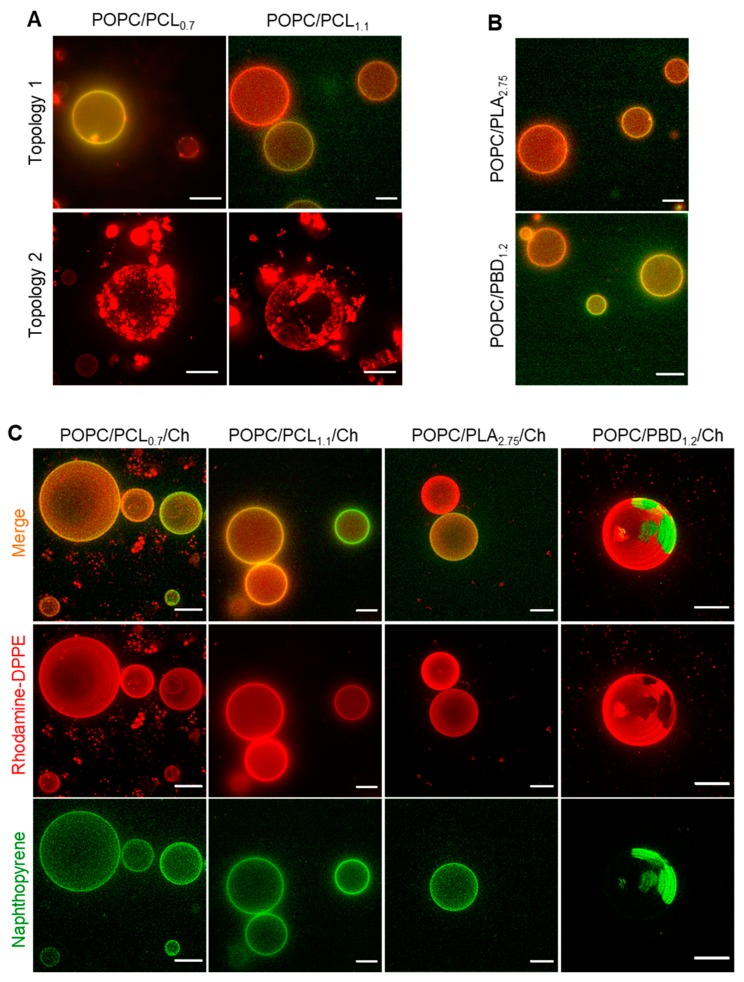
Hybrid giant unilamellar vesicles prepared by electroformation observed using wide-field fluorescence microscopy. (**A**) 1-palmitoyl-2-oleoyl-*sn*-glycero-3-phosphocholine (POPC)/polycaprolactones (PCLs). (**B**) POPC/polylactic acid (PLA_2.75_) and POPC/polybutadiene (PBD_1.2_). (**C**) POPC/BCP/cholesterol (Ch) (1:1:1) labeled with 0.5–1.0 mol% of 1,2-dipalmitoyl-*sn*-glycero-3-phosphoethanolamine-*N*-(lissamine rhodamine B sulfonyl) (Rhod-DPPE) and 1.0–1.5 mol% Naphthopyrene. Scale bars, 10 μm.

Mapping a phase-separated topology is useful to better control the functionality of reconstituted protein in the hybrid bilayer [39,40]. To examine if the hybrid vesicles display a coexisting phase separated topography, we prepared giant unilamellar vesicles (GUVs) by the electroformation method [36]. The vesicles were doped with 1,2-dipalmitoyl-*sn*-glycero-3-phosphoethanolamine-*N*-(lissamine rhodamine B sulfonyl) (Rhod-DPPE), which is known to partition into lipid-rich lamellar phase [41] (Figure 2). 

POPC/PBD_1.2_ and POPC/PLA_2.75_ formed GUVs with homogeneous surface topology comparable to GUVs consisting of POPC alone, indicating that no micron-sized polymer-lipid phase separation has occurred (Figure 2B). For POPC/PCL_0.7_ and POPC/PCL_1.1_, both homogeneous and topologies with areas of condensed dye aggregates and dye-excluded regions were observed (Figure 2A). Based on the liquid-disordered phase preferential of Rhod-DPPE, this suggests that micro-sized phase separation has occurred, albeit with a morphology distinct from the typical phase separation on GUV topology [42], whereby patches of dye-enriched and dye-excluded regions are seen. The dye appeared to segregate into membrane defects, as has been observed on faceted polyhedron vesicles [43]. Studies using Brownian dynamics [44] suggest that the liquid-ordered facets are connected by highly curved fluid regions in the vesicles, which could result in an accumulation of Rhod-DPPE. The faceted topologies for POPC/PCLs may suggest that the local interfacial tension of these vesicles is distinct from the other polymers. Consistent with the inability of the polymers to form the assembly of nano-sized polymersomes, we did not observe any micron-scale vesicles when electroformation was performed, except for PBD_1.2_ (<10 μm, Figure S3). The failure to form vesicular structure may be constrained by the much smaller polymer size used, as larger polymers of the same polymer types are able to form vesicular structures [29,38,45,46].

### 3.4. Surface Topography of Hybrid POPC/BCP/Cholesterol Giant Unilamellar Vesicles

To investigate if polymer-rich and lipid-rich domains can form in the presence of cholesterol (Ch), which has been previously shown to promote polymer-lipid phase separation by condensing fluid phase lipid [23,35], we incorporated cholesterol into the lipid-polymer composition at equimolar ratio, i.e., POPC:BCP:Ch at 1:1:1 mol%, doped with 0.5–1.0 mol% of Rhod-DPPE and 1.0–1.5 mol% of naphthopyrene. As described above, Rhod-DPPE partitions into fluid disordered lipid phase, whereas naphthopyrene partitions into the polymer-rich phase. 

A phase separated surface topology was observed readily for POPC/PBD_1.2_/Ch, where Rhod-DPPE (red in color) and naphthopyrene (green in color) partitioned into mutually exclusive regions (Figure 2C). Green spherical polymer-rich domains were surrounded by interconnected lipid phase, an effect of line tension minimization on a de-mixed fluid phase bilayer [23,47] and a result of lipid chain-ordering effect of cholesterol [23,35]. The biodegradable hybrid GUVs showed homogenous surfaces. For POPC/PCLs/Ch and POPC/PLA/Ch, Rhod-DPPE-dense or excluded regions observed for the cholesterol-depleted GUVs were replaced by a homogeneous one, suggesting that the nanometer-sized defects that may have contributed to dye accumulation (Figure 2A) have vanished in the presence of cholesterol, consistent with the fluidizing effect of cholesterol observed in other studies [23,43]. 

We further explored other compositions of the ternary systems by forming GUVs at POPC/BCP/Ch ratios (Figure S4A) and confirmed the homogeneous topographies for all the compositions tested (Figures S4B and S5). This is in agreement with our hypothesis that a reduced thickness mismatch between lipid and BCP leads to a reduced pre-existing lateral line tension [42], contributing to favorable lipid-BCP interaction. Furthermore, the homogeneous topography is likely associated with the emergence of mixed chain overlap between lipid and BCPs, such that lipid molecules on one leaflet become partially interdigitated with hydrophobic blocks of the BCPs, consistent with observation from the electro micrographs. 

### 3.5. Enzymatic Activity on Hybrid POPC/BCP Large Unilamellar Vesicles

To further investigate the surface properties of these hybrid lipid-polymer assemblies, we monitored the effects of phospholipase on the structural integrity of the hybrid LUVs. We have used the Ca^2+^-dependent, surface-active, secreted phospholipase A_2_ (sPLA_2_) from bee venom (*Apis mellifera*). Briefly, sPLA_2_, which belongs to a superfamily of lipases, catalyzes the hydrolysis of ester bond at the *sn*-2 position of phospholipids, producing free fatty acids and lysophospholipids, and its effects on membrane due to the resultant reaction products have been extensively characterized [48,49,50,51]. The initial interfacial binding of sPLA_2_ with the membrane is mediated by a defined binding surface (*i*-face) on sPLA_2_ [52,53,54]. Hence, the activity of sPLA_2_ enables us to examine nanometer-scale functional lipid motifs. 

**Figure 3:**
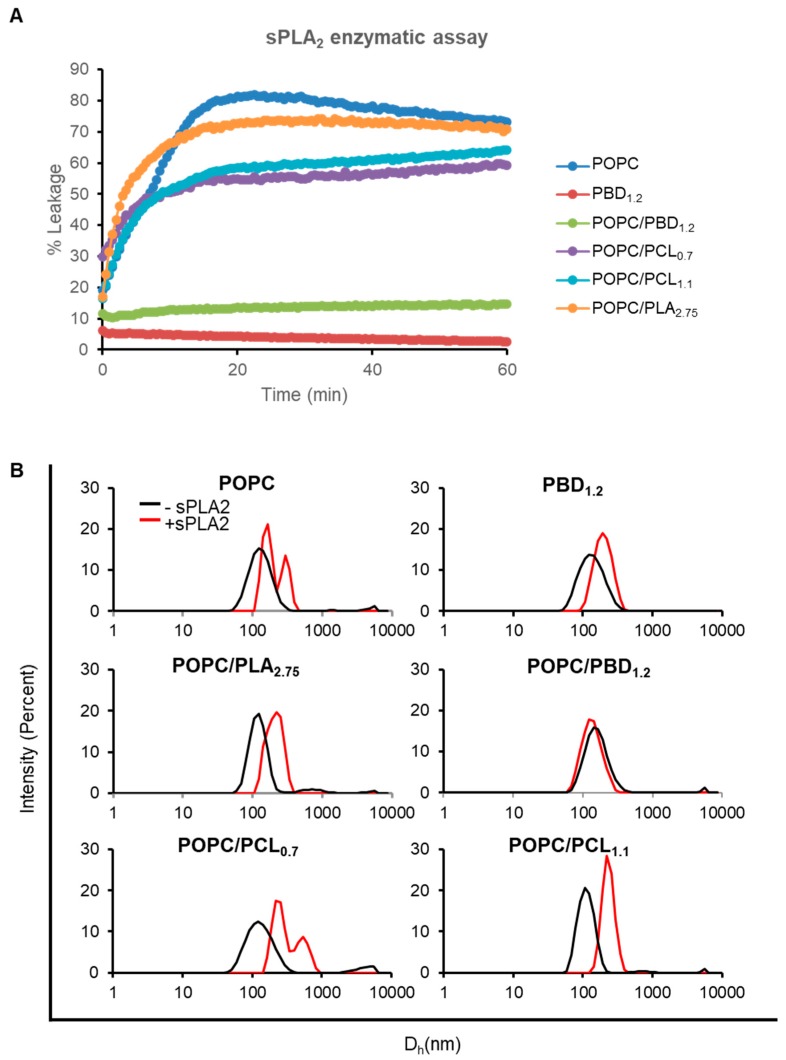
Phospholipase A_2_ (sPLA_2_) enzymatic activity on hybrid large unilamellar vesicles (LUVs). (**A**) Calcein release kinetics for hybrid LUVs subjected to POPC:sPLA_2_ ratio of 100:1 in 1 mM CaCl_2_ at room temperature. (**B**) Intensity-weighted diameter hydrodynamic diameter of hybrid LUVs before and after 1 h sPLA_2_ incubation.

To begin, we examined the sensitivity of the vesicles to sPLA_2_ by using a calcein leakage assay. Vesicles encapsulating calcein were prepared as described above and leakage of calcein serves as a direct readout of sPLA_2_ activity. At sPLA_2_:POPC ratio of 1:100, we detected slightly less leakage (~10% lower) for POPC/PCL_0.7_ and POPC/PCL_1.1_, as compared to POPC after 1 h of enzymatic reaction (Figure 3A). The response of POPC/PLA_2.75_ was similar to that of POPC. At much higher sPLA_2_:POPC ratios (1:50 to 1:12), all vesicles containing biodegradable polymers showed complete leakage (Figure S6). As a negative control, PBD_1.2_ vesicles remained intact (<5% leakage at all sPLA_2_ concentrations tested). Similarly, POPC/PBD_1.2_ was also resistant to enzymatic digestion, consistent with our previous findings [34]. The fluorescence signal detected in a calcein release assay is the net effect of the released calcein from the self-quenched environment and possibly photobleaching effect. The decreasing fluorescence intensity after the maxima (Figure 3A and Figure S6) is observed in cases where near complete leakage has occurred. For vesicles, that have only shown partial response, continuous leakage of calcein may compensate for the photobleaching. As a result, such decreasing trend is not observed. 

In support of the membrane permeability result, we further examined size changes of the vesicles after enzymatic digestion. We performed DLS measurements on LUVs incubated with 100 nM sPLA_2_ for 1 h at which sPLA_2_ reaction was complete and a steady membrane state was reached. Membrane restructuring, if any, could then be measured. POPC, POPC/PLA_2.75_, and POPC/PCLs LUVs that have shown varying degrees of sensitivity to sPLA_2_, exhibited an increase (at least two-fold) in the hydrodynamic diameter (D_h_) (Figure 3B and Figure S7), consistent with the previously reported increase in vesicle size after sPLA_2_ hydrolysis [55,56]. This is attributed to vesicle opening and re-assembly after the emergent of hydrolyzed products–lysophospholipids and fatty acids, which are known to destabilize membrane, either created in situ in a preformed lamellar phase or added externally [48,49,51]. In contrast, POPC/PBD_1.2_ did not show observable size increase. Surprisingly, PBD_1.2_ vesicles showed a slight change in size distribution, likely due to non-specific sPLA_2_ membrane binding. The enzymatic activity on the hybrid SUVs demonstrates that the LUVs retain a dynamic, lipid-like surface topology, even in the presence of 50 mol% of biodegradable polymer. 

### 3.6. Enzymatic Activity on Hybrid POPC/BCP Giant Unilamellar Vesicles

**Figure 4:**
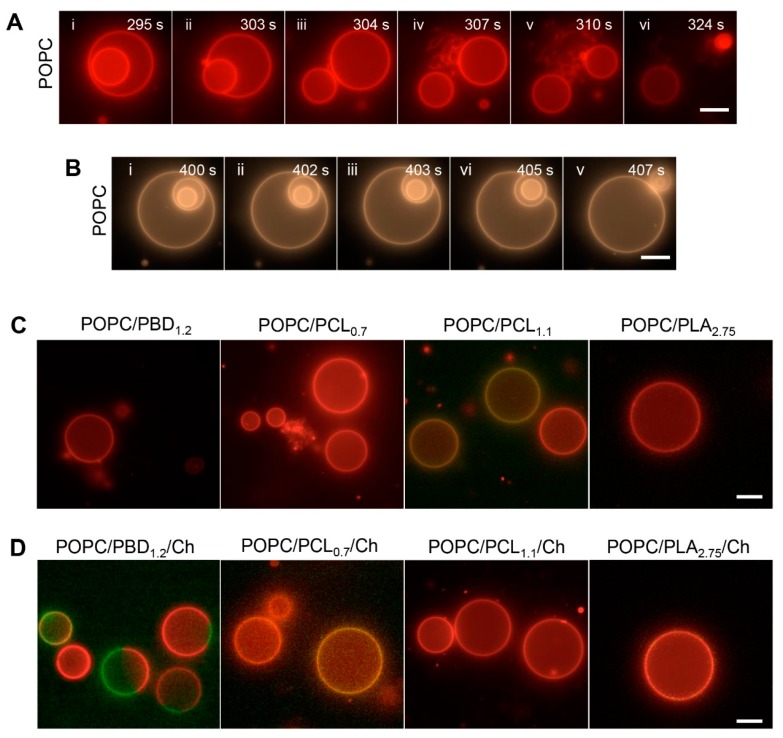
sPLA_2_ enzymatic activity on hybrid giant unilamellar vesicles (GUVs): (**A**,**B**) Morphological transitions of POPC GUVs subjected to 100 nM sPLA_2_ solution, supplemented with 200 nM CaCl_2_. Time-lapse fluorescence images of: (**A**) vesicle expulsion followed by external tubulation and size shrinkage and (**B**) vesicle expulsion followed by appearance of flaccid parent vesicles. (**C**,**D**) Hybrid GUVs subjected to 100 nM sPLA_2_ solution, supplemented with 200 nM CaCl_2_. Images are taken after at least 10 min of sPLA_2_ incubation. POPC/BCP (1:1, mol%) GUVs (**C**). POPC/BCP/Ch (1:1:1 mol%) GUVs (**D**). All hybrid GUVs are supplemented with 0.5–1.0 mol% Rhod-DPPE and 1.0–1.5 mol% 1,2-dipalmitoyl-sn-glycero-3-phosphoethanolamine-N-(7-nitro-2-1,3-benzoxadiazol-4-yl) (NBD-DPPE). Scale bars, 10 μm.

To further understand the membrane effect as a consequence of sPLA_2_ interaction, hybrid GUVs, with or without cholesterol (at 1:1 or 1:1:1 molar ratios, respectively), were prepared. We first characterized and benchmarked sPLA_2_ effects on POPC GUVs. Two major morphological changes were observed. The first was characterized by vesicle size shrinkage (Figure 4A and Video S1). In some instances, formation of membrane tubules was observed (Figure 4A (iv–v)). Ultimately, the digested vesicles shrank to a dense lipid aggregate, indicating that the phospholipids were hydrolyzed, producing lysophospholipids and free acids, which give rise to the instability in the vesicle membrane (Figure 4A (vi)). The second involved spontaneous expulsion of interior vesicles [57] (Figure 4A (ii–iii),B (i–v) and Video S2). 

The hybrid biodegradable GUVs remained topographically intact and spherical, independent of cholesterol (Figure 4C). In comparison, the response of POPC/PBD_1.2_ GUVs to sPLA_2_ was qualitatively different from the hybrid biodegradable GUVs, showing transient membrane flickering. Surprisingly, POPC/PBD_1.2_/Chol GUVs, which showed a phase-separated topology, did not show size shrinkage nor membrane tubulation from the lipid-rich phase. This may suggest that some PBD_1.2_ might be interspersed in the POPC-rich phase and perturb the binding of sPLA_2_ although the majority of PBD_1.2_ were segregated from the POPC-rich region. 

To understand the contrasting effects of sPLA_2_ on POPC GUVs and on GUVs containing biodegradable polymers, a unifying biophysical mechanistic understanding of sPLA_2_ needs to be discussed. Based on a series of theoretical and experimental works [53,57,58,59,60], briefly, when sPLA_2_ hydrolyzes POPC into the two membrane destabilizing compounds, a lysophospholipid and a free fatty acid, their conical shapes perturb the thermodynamic equilibrium of POPC bilayer [48,50,61]. Given sufficient number of conical shaped species in the outer leaflet, external tubules or evaginations will form. In another case, as a consequence of the digestion products, the pore nucleation energy barrier lowers and is more easily overcome, such as when an object from the vesicle interior comes in proximity to the inner monolayer [57] (Figure 4A (ii)). From the time-lapse images, we also observed that during the expulsion of inner vesicle, the outer vesicle remained spherical and tense, driven by both membrane tension and line energy (due to the exposure of hydrophobic tail to solvent) [57] (Figure 4A (iii)). Not all parent vesicles remained tense during the expulsion of inner vesicle. In some instances, the parent vesicle could become flaccid (Figure 4B (vi)), likely due to convection of solute and solvent through nano-scale width surrounding the daughter vesicle, effectively lowering volume-to-area ratio of the parent vesicle. The lack of membrane tubulation and size shrinkage for the hybrid biodegradable GUVs thus suggest only limited enzymatic activity, if any, was present. Comparing the enzymatic response in the case of LUV, this implies that an apparent vesicle size (or membrane curvature and tension) dependency of sPLA_2_ activity on hybrid lipid-polymer assemblies [62], as membrane tension is inversely proportional to vesicle size [63,64]. 

The general reduction in the activity of sPLA_2_ on hybrid GUVs can further be understood based on the availability of a complementary lipid patch to the *i*-face of sPLA_2_. As sPLA_2_ activity on phospholipid is dependent on the availability of a binding surface of around 1500 Å^2^ (equivalent to about 30 POPC molecules) [54,65], existence of nanoscale mixed topology of lipid and polymer [25] likely perturb its binding through height fluctuations at the monomer scale [11,66], masking the accessibility of the enzyme to the *sn*-2 position of phospholipids. This increased stability towards enzymatic digestion is reminiscent of PEGylated-lipid, but with the advantage of forming a vesicular structure at high polymer fraction, i.e., 50 mol% in the current study as compared to an upper limit of 10 mol% for PEGylated lipid before transforming into a micellar form [67]. Furthermore, considering that micellar states are not detected after sPLA_2_ digestion, rather an increase in size is observed (Figure 3B), it suggests that the vesicles are not completely broken down but are reorganized, during which its content leaks. The reorganization observed for the nano-scale vesicles may be related to the difference in membrane tension and the nuance difference in the chemical structure of the BCPs on the hybrid vesicle surface, which requires further extensive investigation.

## 4. Conclusions

We have prepared nano- and micron-scale hybrid lipid-polymer vesicles consisting of phospholipids and low M_W_ biodegradable BCPs. Self-assembly of the low M_W_ BCPs is promoted by facile mixing with POPC, as the BCPs do not form closed lamellar structures on their own. The hybrid vesicles possess a bilayer thickness similar to that of natural lipid membrane and are able to encapsulate hydrophilic small molecules. The surface topographies of these vesicles are lipid-like as they are responsive to enzymatic digestion, which can be modulated by changing the polymer types. While we have shown here that with POPC, other phospholipid types should, in principle, be useful in exploiting this phospholipid-assisted hybrid vesicle forming strategy and contribute to designing a larger repertoire of functional vesicles for future applications.

## Figures and Tables

**Table 1:** Chemical compositions of formulations used in this study and molecular characteristics of the block copolymers.

Formulation.	Composition	POPC:BCP (mol:mol)	Mol. Wt (g/mol)			*f_hydrophilic_*	PDI
			M_n_	M_A_	M_B_		
*Lipid*							
**POPC**	POPC	1:0	760				
*BCP*							
**PCL_0.7_**	PEG_12_-PCL_6_	0:1	1250	550	700	0.44	1.13
**PCL_1.1_**	PEG_12_-PCL_9_	0:1	1650	550	1100	0.33	1.17
**PLA_2.75_**	PEG_16_-PLA_38_	0:1	3450	700	2750	0.20	1.12
**PBD_1.2_**	PEG_13_-PBD_22_	0:1	1800	600	1200	0.33	1.09
*Hybrid (POPC/BCP or POPC/BCP/Ch)*							
**POPC/PCL_0.7_**	POPC: PEG_12_-PCL_6_	1:1					
**POPC/PCL_1.1_**	POPC: PEG_12_-PCL_9_	1:1					
**POPC/PLA_2.75_**	POPC: PEG_16_-PLA_38_	1:1					
**POPC/PBD_1.2_**	POPC: PEG_13_-PBD_22_	1:1					
**POPC/PCL_0.7_/Ch**	POPC: PEG_12_-PCL_6_: Cholesterol	1:1:1					
**POPC/PCL_1.1_/Ch**	POPC: PEG_12_-PCL_9_: Cholesterol	1:1:1					
**POPC/PLA_2.75_/Ch**	POPC: PEG_16_-PLA_38_: Cholesterol	1:1:1					
**POPC/PBD_1.2_/Ch**	POPC: PEG_13_-PBD_22_: Cholesterol	1:1:1					

The total molecular weight of block copolymers (BCPs) mentioned here is in the number average molecular weight denoted as M_n_, M_A_, and M_B_ refer to number average molecular weight of hydrophilic and hydrophobic blocks of the BCP, respectively. PDI = Polydispersity Index of BCPs.

## Supplementary Materials

The following are available online at https://www.mdpi.com/2073-4360/12/4/979/s1. Figure S1: Morphology of BCPs. Cryo-TEM images of PCL_0.7_ (A), PCL_1.1_ (B), and PLA_2.75_ (C). Average membrane thickness is indicated in the bottom left inset of each image. (D) Intensity-weighted hydrodynamic diameter of the three BCPs. Figure S2: Calcein encapsulation of the hybrid vesicles. Raw fluorescence intensity values recorded for the vesicles (Control) after dialysis and the Triton-treated vesicles (TX-100) are presented. (A) Vesicles samples after dialysis of 24 h. (B) Vesicle samples after dialysis for 48 h. Figure S3: Giant unilamellar vesicles (GUVs). PBD_1.2_ GUVs, doped with 1% of naphthopyrene. GUVs were produced by electroformation method. Figure S4: Hybrid giant unilamellar vesicles (GUVs). (A) Ternary phase diagram depicting the POPC/BCP/Cholesterol compositions studied. (B) Hybrid GUVs consisting of POPC/PCL_0.7_/Cholesterol at different molar ratios, doped with 0.5–1.0 mol% of Rhod-DPPE and 1.0–1.5 mol% of naphthopyrene. GUVs were produced by the electroformation method. Scale bar, 10 μm. Figure S5: Hybrid GUVs consisting of POPC/PCL_1.1_/Cholesterol (A) and POPC/PCL_2.75_/Cholesterol (B) at different mol%, doped with 0.5–1.0 mol% of Rhod-DPPE and 1.0-1.5 mol% of naphthopyrene. Formulations were produced by electroformation method. Figure S6: sPLA2 enzymatic activity on hybrid large unilamellar vesicles (LUVs). Calcein release kinetics for hybrid LUVs subjected to POPC:sPLA_2_ at the maximum tested ratios of 25:1 and 12:1 for POPC and hybrid vesicles, respectively, in 1 mM CaCl_2_ at room temperature. Figure S7: sPLA_2_ enzymatic activity on hybrid large unilamellar vesicles (LUVs). (A) Intensity-weighted hydrodynamic diameter of all formulations before (solid lines) and after (dotted lines) sPLA_2_ treatment. (B) Intensity weighted size distribution, z-average, and polydispersity (PDI) comparision between fomulations presence and absence of sPLA_2_. Representative result highlighting variability between technical replicates. Video S1: sPLA_2_ enzymatic activity on POPC giant unilamellar vesicles (GUVs). This video shows vesicle expulsion followed by external tubulation and vesicle size shrinkage. GUVs are subjected to 100 nM sPLA_2_ with 200 nM CaCl_2_ solution. Vesicles are supplemented with 0.5–1.0 mol% Rhod-DPPE and 0.5–1.0 mol% 1,2-dipalmitoyl-*sn*-glycero-3-phosphoethanolamine-*N*-(7-nitro-2-1,3-benzoxadiazol-4-yl) (NBD-DPPE). Scale bars, 10 μm. Video S2: sPLA_2_ enzymatic activity on POPC giant unilamellar vesicles (GUVs). This video shows vesicle expulsion followed by appearance of flaccid parent vesicles. GUVs are subjected to 100 nM sPLA_2_ with 200 nM CaCl_2_ solution. Vesicles are supplemented with 0.5–1.0 mol% Rhod-DPPE and 0.5–1.0 mol% NBD-DPPE. Scale bars, 10 μm.
