# Supplementary material for: Facile Mixing of Phospholipids Promotes Self-Assembly of Low-Molecular-Weight Biodegradable Block Co-Polymers into Functional Vesicular Architectures
Source: Polymers (Basel). 2020 Apr 22;12(4):979. doi: 10.3390/polym12040979 (PMC7240622; doi:10.3390/polym12040979)
Supplement: Supplementary file 1 [file polymers-12-00979-s001.zip › polymers-745654-suppl-v3.docx]

**Supplementary Materials**

Facile mixing of phospholipids promotes self-assembly of low-molecular-weight biodegradable block co-polymers into functional vesicular architectures

Amit Kumar Khan ^1,2^, James Ho CS ^1^, Susmita Roy ^1^, Bo Liedberg ^1^ and Madhavan Nallani ^1,2^*

^1^ Centre for Biomimetic Sensor Science, School of Materials Science and Engineering, Nanyang Technological University, 50 Nanyang Drive 637553, Singapore; [mnallani@ntu.edu.sg](mailto:mnallani@ntu.edu.sg)

^2^ ACM Biolabs Pte Ltd, NTU Innovation Center, 71 Nanyang Drive, S638075 Singapore; [mnallani@acmbiolabs.com](mailto:mnallani@acmbiolabs.com)

***** Correspondence: [mnallani@ntu.edu.sg](mailto:mnallani@ntu.edu.sg); Tel.: +65-62655646

Received: date; Accepted: date; Published: date


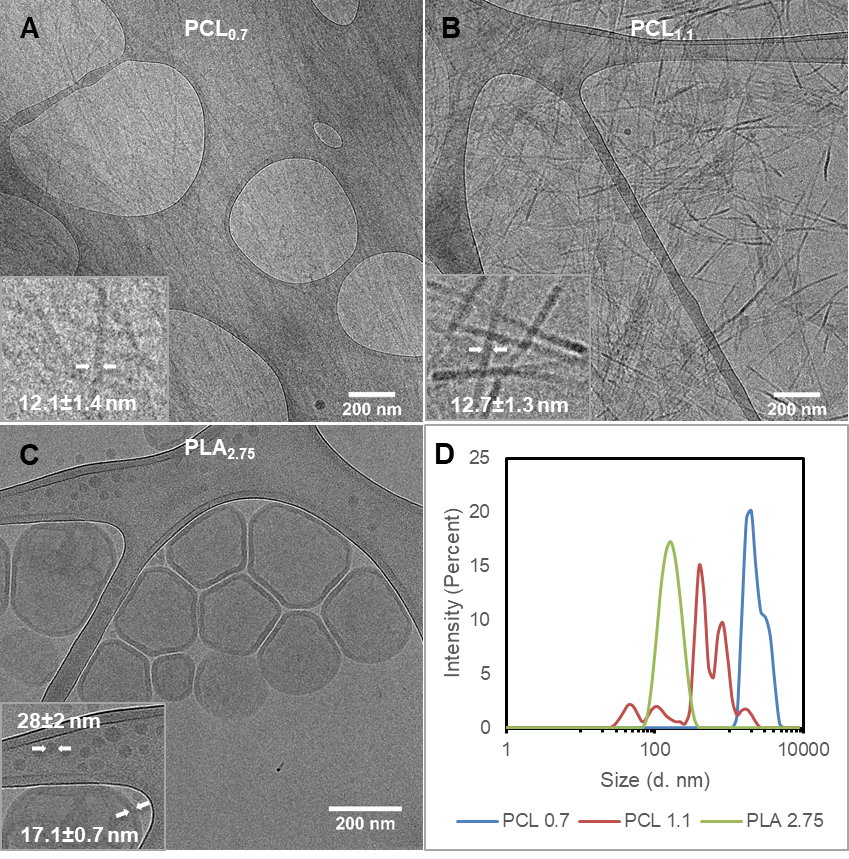


**Figure S1**. **Morphology of BCPs**. Cryo-TEM images of PCL_0.7_ (**A**), PCL_1.1_ (**B**), and PLA_2.75_ (**C**). Average membrane thickness is indicated in the bottom left inset of each image. (**D**) Intensity-weighted hydrodynamic diameter of the three BCPs.


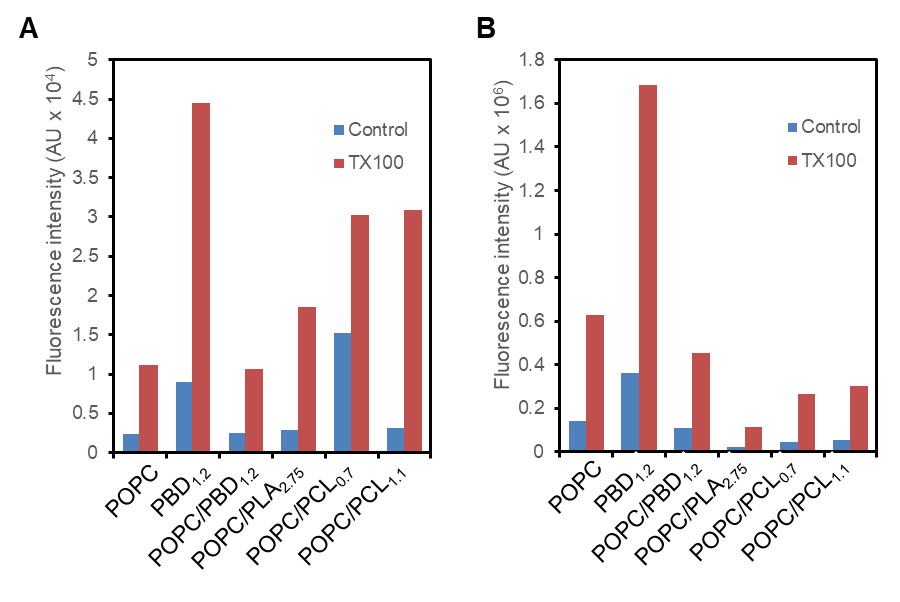


**Figure S2 Calcein encapsulation of the hybrid vesicles**. Raw fluorescence intensity values recorded for the unruptured vesicles (Control) and the Triton-treated vesicles (TX100) are presented. (A) Vesicles samples dialyzed for 24 h. (B) Vesicle samples dialyzed for 48 h.


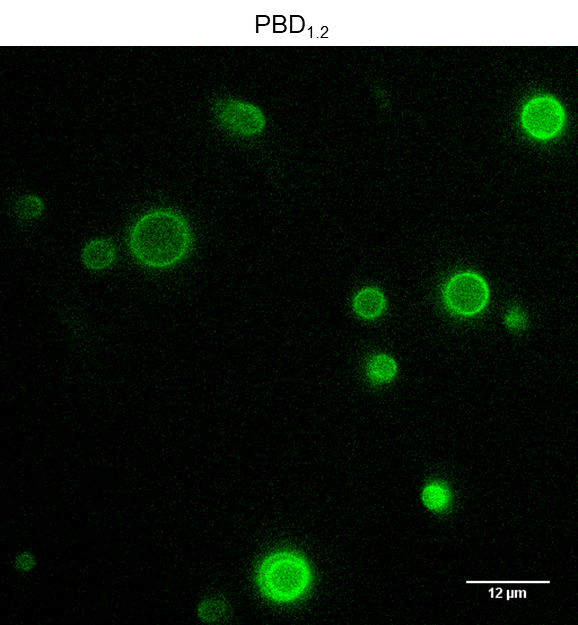


**Figure S3. Giant unilamellar vesicles (GUVs)**. PBD_1.2_ GUVs, doped with 1% of Naphthopyrene. GUVs were produced by electroformation method.


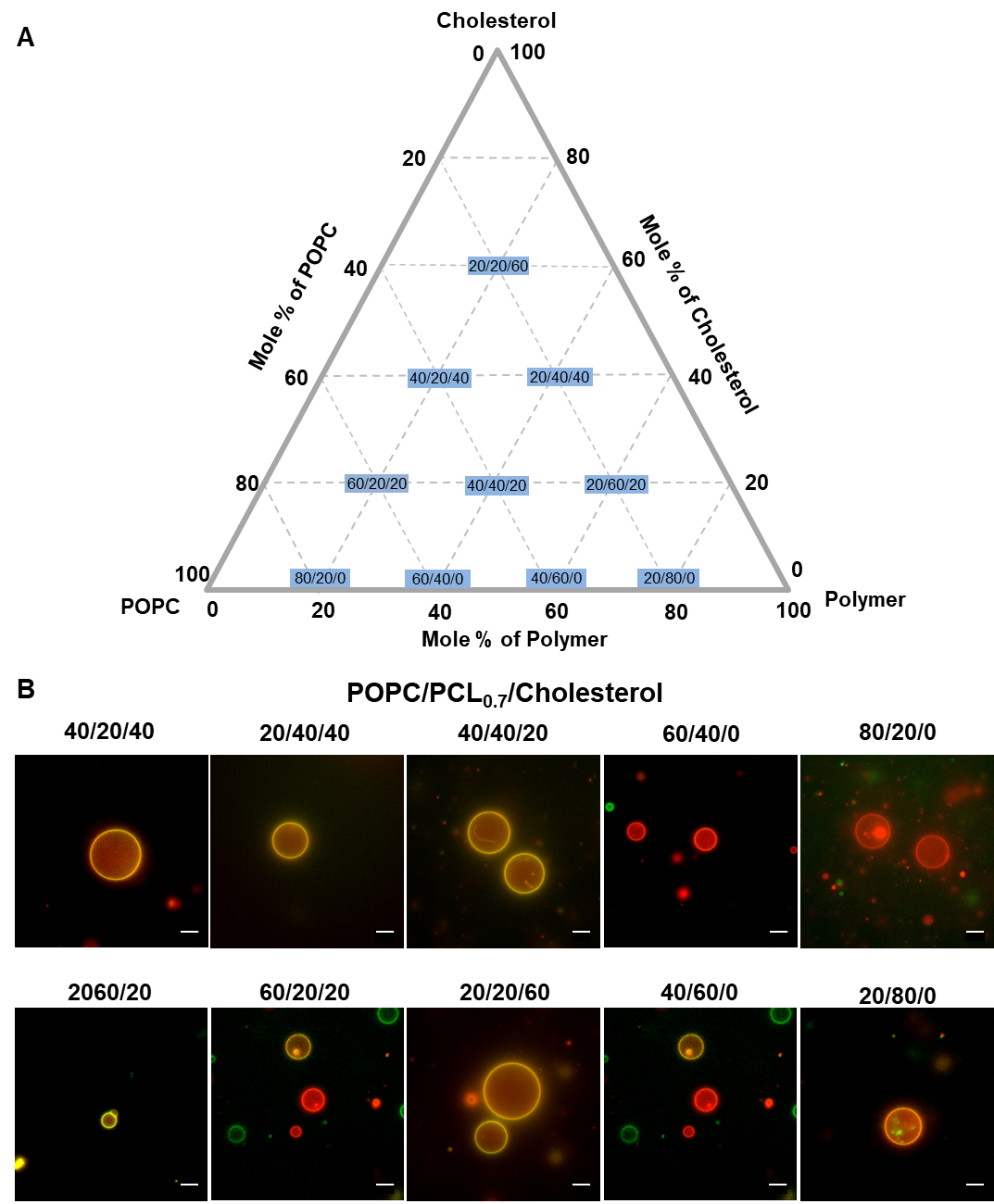


**Figure S4. Hybrid giant unilamellar vesicles (GUVs).** (**A**) Ternary phase diagram depicting the POPC/BCP/Cholesterol compositions studied. (**B**) Hybrid GUVs consisting of POPC/PCL_0.7_/Cholesterol at different molar ratios, doped with 0.5-1.0 mol% of Rhod-DPPE and 1.0-1.5 mol% of naphthopyrene. GUVs were produced by the electroformation method. Scale bars, 10 μm.


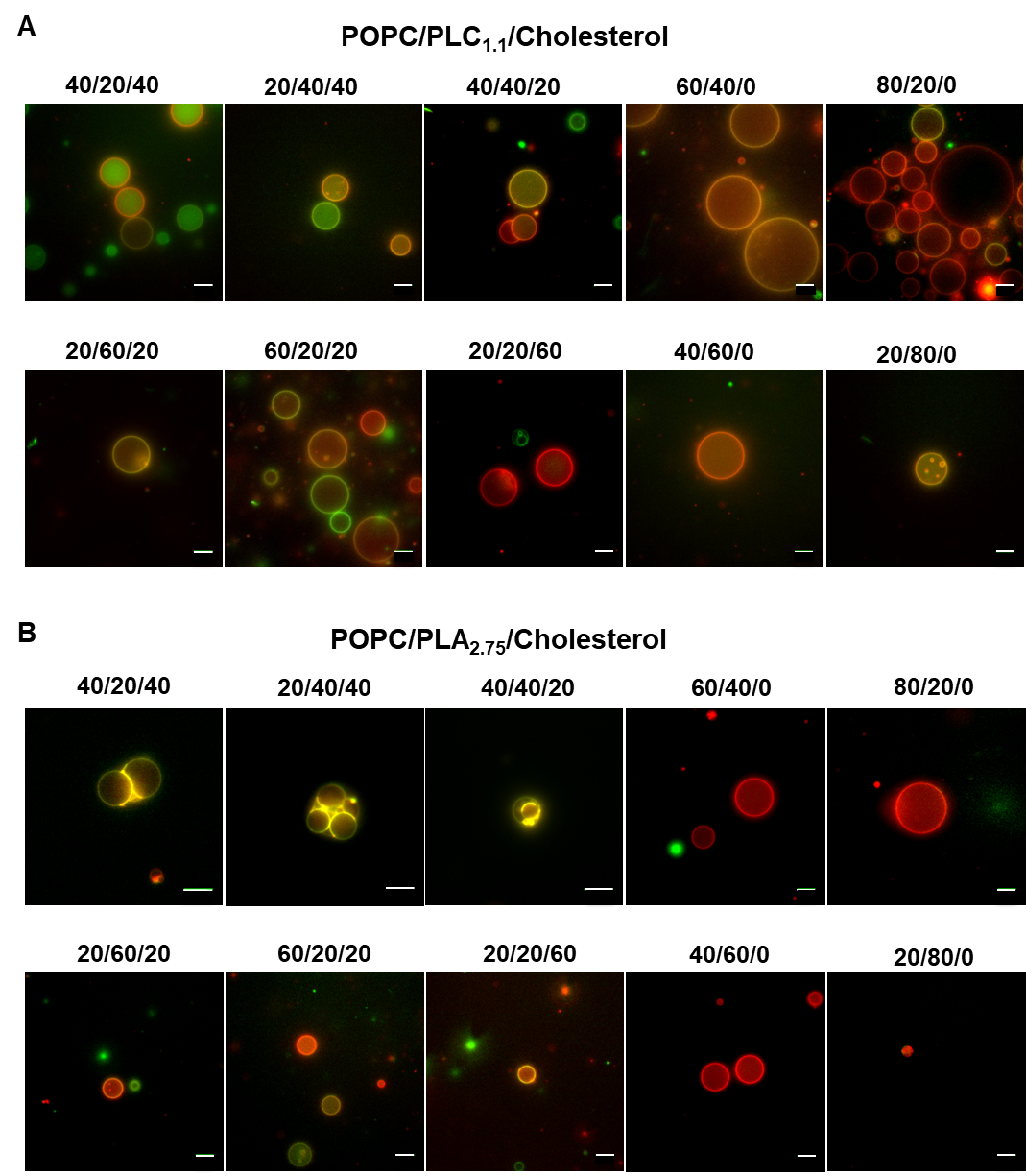


**Figure S5**. Hybrid GUVs consisting of POPC/PCL_1.1_/Cholesterol (**A**) and POPC/PLA_2.75_/Cholesterol (**B**) at different mol%, doped with 0.5-1.0 mol% of Rhod-DPPE and 1.0-1.5 mol% of naphthopyrene. Formulations were produced by the electroformation method. Scale bars, 10 μm.

**
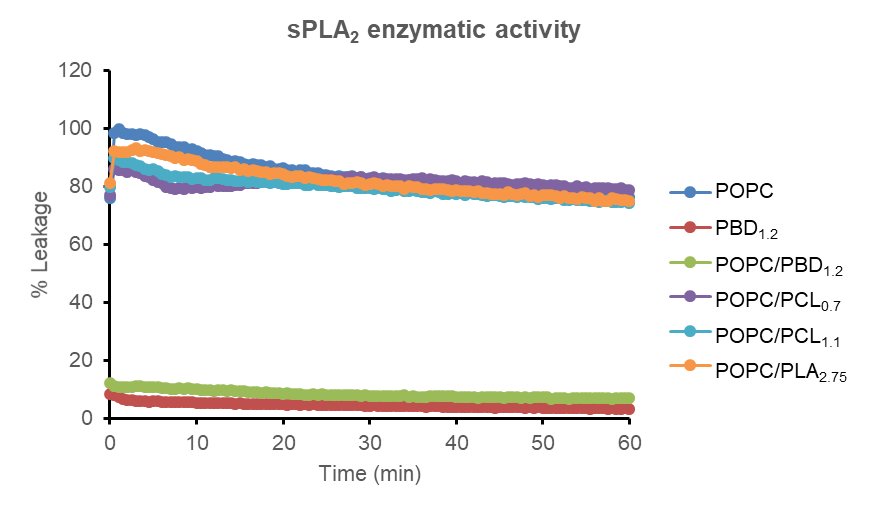
**

**Figure S6. sPLA_2_ enzymatic activity on hybrid large unilamellar vesicles (LUVs)**. Calcein release kinetics for hybrid LUVs subjected to POPC:sPLA_2_ at the maximum tested ratios of 25:1 and 12:1 for POPC and hybrid vesicles, respectively, in 1 mM CaCl_2_ at room temperature.


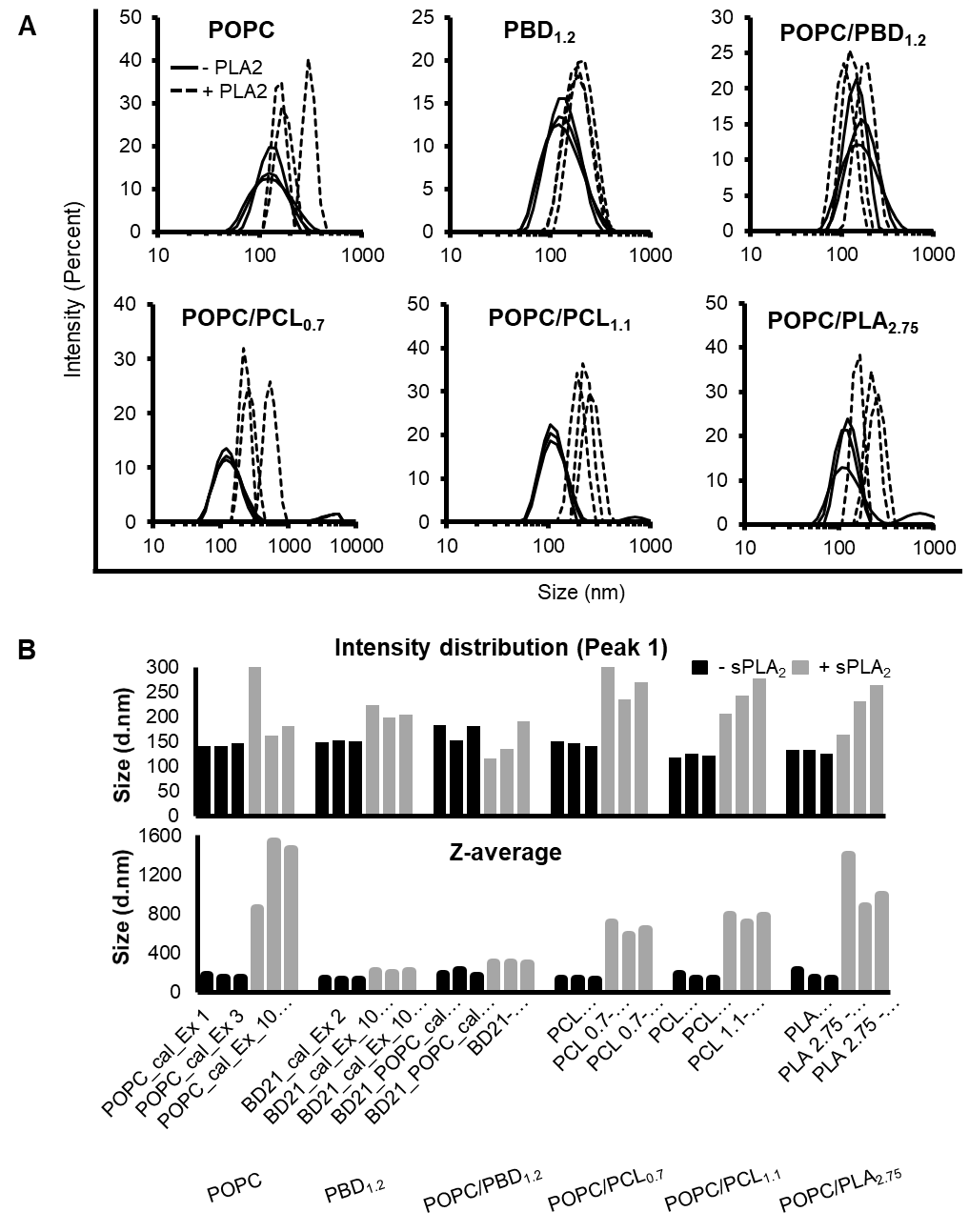


**Figure S7. sPLA_2_ enzymatic activity on hybrid large unilamellar vesicles (LUVs).** (**A**) Intensity-weighted hydrodynamic diameter of all formulations before (solid lines) and after (dotted lines) sPLA_2_ treatment. (**B**) Intensity weighted size distribution, z-average, and polydispersity (PDI) comparision between fomulations presence and absence of sPLA_2_. Representative result highlighting variability between technical replicates.

**Video S1. sPLA_2_ enzymatic activity on POPC giant unilamellar vesicles (GUVs). This video shows** vesicle expulsion followed by external tubulation and vesicle size shrinkage. GUVs are subjected to 100 nM sPLA_2_ with 200 nM CaCl_2_ solution. Vesicles are supplemented with 0.5-1.0 mol% Rhod-DPPE and 0.5-1.0 mol% NBD-DPPE. Scale bars, 10 μm.

**Video S2. sPLA_2_ enzymatic activity on POPC giant unilamellar vesicles (GUVs). This video shows** vesicle expulsion followed by appearance of flaccid parent vesicles. GUVs are subjected to 100 nM sPLA_2_ with 200 nM CaCl_2_ solution. Vesicles are supplemented with 0.5-1.0 mol% Rhod-DPPE and 0.5-1.0 mol% NBD-DPPE. Scale bars, 10 μm.

| 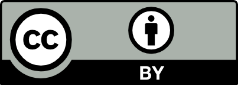 | © 2020 by the authors. Submitted for possible open access publication under the terms and conditions of the Creative Commons Attribution (CC BY) license (http://creativecommons.org/licenses/by/4.0/). |
| --- | --- |
